# Characteristics, risk factors and outcome of BKV nephropathy in kidney transplant recipients: a case–control study

**DOI:** 10.1186/s12879-023-08043-z

**Published:** 2023-02-06

**Authors:** Julien Gras, Arnaud Le Flécher, Axelle Dupont, Jérôme Vérine, Ali Amara, Constance Delaugerre, Jean Michel Molina, Marie Noëlle Peraldi

**Affiliations:** 1 Infectious Disease Department, APHP-Saint Louis Hospital, 1 Avenue Claude Vellefaux, Paris, France; 2 INSERM U944, «Biology of Emerging Viruses» Team, Institut de Recherche Saint Louis, APHP-Saint Louis Hospital, Paris, France; 3 Université Paris Cité, Paris, France; 4 Nephrology and Kidney Transplant Department, APHP-Saint Louis Hospital, Paris, France; 5 Biostatistics and Medical IT Department, APHP-Saint-Louis Hospital, Paris ECSTRA Team, UMR 1153 INSERM, Paris Diderot University, Sorbonne Paris Cité, Paris, France; 6 Pathology Department, APHP-Saint Louis Hospital, Paris, France; 7 Virology Department, APHP-Saint Louis Hospital, Paris, France

**Keywords:** BK virus, BKV associated nephropathy, Kidney transplantation, Risk factors

## Abstract

**Background:**

Following kidney transplantation, BK virus associated nephropathy (BKVN) occurs in 1 to 10% of kidney transplant recipients (KTR) and represents a major cause of graft loss. We aim at identifying factors associated with biopsy proven BKVN among KTR.

**Methods:**

We conducted a retrospective case–control study including all KTR with a biopsy-proven diagnosis of BKVN between 2005 and 2019. Clinical characteristics and outcome were described. For each case, one control KTR without BKV infection was identified and matched by age, transplant date, and donor status. Factors associated with BKVN diagnosis were identified using exact conditional logistic regression. Comparative survival was described using Kaplan–Meier estimator.

**Results:**

Sixty-four cases of BKVN were identified among 1737 new kidney transplantation (3.7% prevalence). Clinical characteristics did not differ between groups, except for a higher c-PRA among cases. BKVN occurred in a median time of 11 (5–14.5) months after KT, and was associated with a significantly impaired graft function at diagnosis. Following BKVN, 61 (95%) of the patients had immunosuppression reduction, which led to BKV DNAemia resolution in 49% of cases. In multivariate analysis, factors associated with BKVN diagnosis were lymphopenia < 500/mm^3^ and a prednisone dose > 7.5 mg/day. Median duration of follow-up was 40 months for both groups. BKVN was associated with a significantly increased risk of graft rejection (*P* = 0.02) and return to dialysis (*P* = 0.01).

**Conclusions:**

BKVN remains a severe complication in KTR and is associated with an increased risk for acute rejection and return to dialysis. Lymphopenia below 500/mm^3^ and corticosteroid maintenance therapy are significantly associated with biopsy-proven BKVN diagnosis.

## Background

BK virus (BKV) is an opportunistic pathogen of the *Polyomaviridae* family, whose reactivation among kidney transplant recipients (KTR) can lead to the development of a tubulo-interstitial nephropathy [1, 2]. BKV associated nephropathy (BKVN) most frequently occurs during the first year following kidney transplantation (KT), and its incidence has increased over the past decades mainly due to the implementation of highly immunosuppressive medications such as the association of calcineurin inhibitors (CNI) and mycophenolic acid (MMF) [3]. Despite the improvement of screening methods based on the regular monitoring of BKV viral load in plasma, BKVN incidence has remained stable over the past years [4]. Following KT, BKV DNAemia is detected in 10 to 20% of kidney transplant recipients, and occurs mostly during the first twelve months [5, 6]. In the absence of any therapeutic intervention, 10 to 50% of the viremic subjects progress to BKVN in a median delay of 2 to 6 weeks, and may result in graft loss [7]. There is currently no specific antiviral drug available, and the mainstay of therapy for significant BKV replication is reducing immunosuppressive drugs [8], which leads to the resolution of BKV DNAemia in up to 80 to 100% of the cases [9]. However, following immunosuppression reduction, T-cell mediated and/or antibody mediated rejection occurs in 4 to 15% of the cases, and can also increase the risk of graft loss [10, 11].

A large number of factors have been associated with an increased risk for BKV infection in KTR including demographic features (older age, male sex, ethnicity) [12, 13], transplantation-related characteristics (deceased donor, ureteral stenting, acute rejection episode) [14–16], and most of all immunosuppressive treatment in particular tacrolimus and/or corticosteroid based regimens [13, 17]. However, in the majority of these studies, BKV infection was defined by the occurrence of BKV DNAuria and/or DNAemia, and only a small number have focused on biopsy-proven BKVN [18]. Moreover, due to the low frequency of this complication, most of these studies had small sample sizes and thus lacked statistical power.

We therefore conducted a retrospective case–control study to describe the clinical features and prognosis of biopsy-proven BKVN in a cohort of kidney transplant recipients, and identify potential factors associated with BKVN diagnosis in this population.

## Methods

### Study design and population

We conducted a case–control study in the APHP (Assistance Publique des Hôpitaux de Paris)—Saint Louis Hospital (Paris, France) on all the kidney transplant recipients who underwent allograft biopsy between January 2005 and December 2018. All patients provided written informed consent for the use of their samples for research purpose at the time of registration on the transplant waiting list. The study was approved by the local ethical comity of the Assistance Publique des Hôpitaux de Paris according to the declaration of Helsinki.

After a systematic screening of local transplant and histological databases, all KTR with a biopsy-proven diagnosis of BKVN according to the Banff Working Group [19] were included: (1) presence at a various degree of the following histological lesions: viral cytopathic changes, interstitial inflammation, tubular atrophy, and interstitial fibrosis; (2) positive staining for SV40 LTag on immunohistochemistry (IHC). For each BKVN case, one control KTR who did not develop BKV infection following transplantation (neither BKVN nor BKV DNAemia) was randomly selected, and matched according to the following criteria: (1) age (± 3 years); (2) date of transplantation (± 1 year); (3) donor type (deceased or living donor).

### Kidney transplantation protocol and follow-up

During kidney transplantation, ureteral stents were placed in all patients and removed 4 weeks later. In accordance with local institutional protocols, induction therapy was based on anti-thymocyte globulins (ATG), except for patients with either HIV infection or past long-term immunosuppression who received basiliximab. ATG (1.5 mg/kg of ideal body weight) was initiated during the surgical procedure and subsequent doses given daily for 7 days. Patients treated with basiliximab were administered 2 doses of 20 mg, one before transplantation and the second on postoperative day 4. All patients received 500 mg methylpredinosolone during KT. Maintenance therapy consisted either in a dual therapy associating a CNI (tacrolimus or ciclosporin A) and MMF, or a triple therapy with the addition of corticosteroids for the other patients. In addition, patients received valganciclovir for CMV prophylaxis (900 mg daily for 3 months in R + or 6 months in D + /R-) and trimethoprim/sulfamethoxazole (400/80 mg daily for 6 months).

Since 2005, all KTR in our center have been screened by PCR for BKV infection on plasma monthly for the first 6 months post-transplant, then every 3 months until the end of year 2. Protocol kidney biopsies were performed on day 0, months 3 and 12 after KT, and in the additional following cases: de novo acute kidney injury or significant proteinuria, suspicion of acute rejection or BKVN. All biopsies were evaluated for evidence of BKVN and acute rejection according to Banff criteria [19], and IHC staining for SV40 LTag performed in case of interstitial inflammation and/or concomitant positive BKV DNAemia.

### Data collection

Each patient’s medical record was reviewed to collect the following data using an electronic standard case report form: (1) demographics (age, sex, ethnicity, primary kidney disease, and underlying comorbidities such as diabetes mellitus); (2) kidney transplant specifics: duration of pre-transplantation dialysis (months), CMV serostatus, donor source, calculated Panel Reactive Antibody (cPRA), HLA mismatch, and immunosuppressive regimen (induction and maintenance therapies). The occurrence of biopsy-proven rejection episodes and/or plasma CMV replication following KT were also recorded.

For each case of BKVN, the date and value of first BKV DNAemia (log_10_IU/ml) was identified using local virologic database. At the time of BKVN diagnosis, the following clinical and laboratory data were collected: immunosuppressive regimen including daily prednisone dose (mg per day, at the exclusion of solumedrol pulses), plasma BK viral load (log_10_IU/ml), serum creatinine level (µmol/L), lymphocyte count 30 days before (/mm^3^) and gamma-globulin level (g/L). For BKVN cases, available stored samples of the diagnostic biopsy in the pathology database were retrieved for re-examination and histological staging of the BKVN according to the ATS classification by a senior pathologist [20]. For matched controls, clinical and laboratory data were also collected at the index date, corresponding to the date for the case being diagnosed with BKVN.

All patients were followed-up from BKVN diagnosis (for cases) or the index date (for controls) until November 30th 2019, and the following events recorded: biopsy-proven acute graft rejection, de novo Donor Specific Antibody (DSA) appearance (with mean fluorescence intensity [MFI] > 3000), return in dialysis, and death.

### Statistical analysis

Continuous variables are presented as medians and interquartile ranges (IQR) and categorical variables as numbers and percentages. Comparison between cases and controls was done using two-sided Student’s t-test for continuous variables, and Chi-2 tests for categorical ones (or Fischer’s test if conditions were not present). Factors associated with the occurrence of biopsy-proven BKVN were identified using univariate exact conditional logistic regression. Only the variables with a *P*-value below 0.1 in the univariate analysis (and the rank of kidney transplant) were included in a multivariate model. Identified are presented in terms of odd ratios (OR) and their 95% confidence interval (95% CI). Survival and event-free survival curves were obtained using Kaplan–Meier plots with censoring for loss to follow-up or end of observation. Comparison between BKVN cases and matched controls was made using log-rank tests. All statistical tests were two-sided and p-values of < 0.05 were considered to be significant. Analyses were performed using R software version 3.6 (http://www.R-project.org).

## Results

### Study population

From January 2005 to November 2019, 64 cases of biopsy-proven BKVN were identified among kidney transplant recipients in our center. These 64 patients had received a kidney transplantation between January 2004 and December 2018. During the same period, 1737 KT were performed, leading to a frequency of BKVN of 3.7%. Sixty-four controls without BKV infection were randomly selected in the KTR cohort, and matched in a 1:1 ratio by age, date of transplant and donor status.

Baseline characteristics of BKVN cases and matched controls are described in Table 1. Both groups were comparable regarding age, sex, ethnicity, or primary kidney disease. Twenty (31%) cases and 14 (22%) controls had been exposed to immunosuppressants before transplantation, due to prior KT or for the treatment of their primary kidney disease (*P* = 0.32, non-significant). KT specifics did not differ between the two groups except for the median c-PRA score which was significantly higher among BKVN cases (50 [IQR: 6–90]) compared to controls (12.5 [IQR: 0–50]; *P* = 0.002). During the transplant surgery, a ureteral stent was placed in all patients for a median duration of 31 (IQR: 24–46) days (no significant difference between the two groups). Following KT, delayed graft function occurred significantly more frequently among control patients (n = 23, 36%) compared to BKVN cases (n = 11, 17%; *P* = 0.028). Eighteen patients developed acute rejection (3 cellular-mediated, 12 antibody-mediated and 3 mixed rejection) with similar incidence between the two groups. BKVN cases were more frequently exposed to solumedrol pulses, plasma exchange, or anti-CD20 for the treatment of acute rejection, although the difference was not statistically different compared to controls.

**Table 1:** Characteristics of the 64 cases of BKV associated nephropathy and matched controls

Variable	BKVN cases (N = 64) *n* (%); median [IQR]	Controls (N = 64) *n* (%); median [IQR]	*p*-value
*Demographic features*			
Age at the time of KT (years)	53 [43–61]	52 [43–61]	0.88
Male sex	37 (58)	45 (70)	0.2
Ethnicity			0.36
Caucasian	34 (53)	26 (41)	
African	25 (39)	30 (47)	
Other *	5 (8)	8 (12)	
Primary kidney disease			0.36
Vascular and/or diabetes	20 (31)	21 (33)	
Chronic glomerulonephritis	19 (30)	19 (30)	
Interstitial nephropathy	7 (11)	6 (9)	
Other**	18 (28)	18 (28)	
Diabetes mellitus	20 (31)	19 (30)	0.92
*Characteristics of KT*			
Duration of pre-transplantation dialysis *(months)*	43.5 [12–72]	47.5 [22–81]	0.47
CMV serostatus donor / recipient (NA = 1)			0.85
D + /R−	8 (13)	5 (8)	
D−/R−	5 (8)	5 (8)	
D + /R +	33 (52)	34 (54)	
D−/R +	17 (27)	19 (30)	
First transplantation	50 (78)	55 (87)	0.49
Deceased donor	51 (80)	51 (80)	1
c-PRA score	50 [6–90]	12.5 [0–50]	**0.002**
HLA mismatch	4 [3–4.5]	4 [2–4]	0.26
Cold ischemia time *(hours)*	14 [7–16]	14 [8–20]	0.54
Delayed graft function	11 (17)	23 (36)	**0.028**
Induction therapy			0.98
ATG	63 (98)	62 (97)	
Basiliximab	1 (2)	2 (3)	
Maintenance therapy			0.26
Dual therapy	17 (27)	23 (36)	
Triple therapy	47 (73)	41 (64)	
*Events between KT and BKVN diagnosis (or index date for controls)*			
Acute rejection episode	11 (17)	7 (11)	0.43
Antibody-mediated	8	4	
Cellular-mediated	1	2	
Mixed rejection	2	1	
Treatment for acute rejection			
Solumedrol pulses	10 (16)	7 (11)	0.6
IV Immunoglobulin administration	5 (8)	6 (9)	1
Plasma exchange	9 (14)	6 (9)	0.58
Anti-CD20	6 (9)	4 (6)	0.74
Plasma CMV reactivation	36 (58)	29 (46)	0.77
*Data at BKVN diagnosis (or index date for controls)*			
Immunosuppressive regimen			
Corticosteroid	48 (75)	42 (66)	0.33
Median dose *(mg/day)*	10 [5–10]	5 [0–10]	**0.005**
Mycophenolic acid	63 (98)	64 (100)	0.96
Tacrolimus	49 (77)	45 (70)	0.55
Ciclosporin	13 (20)	16 (25)	0.67
m-TOR inhibitors	2 (3)	2 (3)	1
Biological data			
Serum creatinine level (µmol/L)	205 [142–269]	126 [100–159]	** < 0.0001**
Lymphocyte count (/mm^3^)	425 [300–700]	585 [368–950]	0.12
Gamma-globulin level (g/L)	9 [7–11]	9.2 [7–11]	0.63

*p* < 0.05 values are statistically significant and shown in bold

*ATG* anti-thymocyte globulin, *BKVN* BK-virus associated nephropathy, *CMV* cytomegalovirus, *c-PRA* calculated panel reactive antibody, *DSA* donor-specific antibodies, *HLA* human leucocyte antigen, *IQR* interquartile range, *IV* intra-venous, *KT* kidney transplantation, *NA* missing data

^*^Caribbean (N = 3, 5% in cases / N = 6, 9% in controls), Asian (N = 2, 3% in both groups)

^**^Undetermined (N = 12, 19%), polycystic kidney disease (N = 6, 9%) in both groups

### Characteristics of BKVN cases

The diagnosis of BKVN was made in a median time of 11 months (IQR, 5–14.5) after KT, and 4 months (IQR, 1–7) after the onset of BK viremia. Immunosuppressive regimen was comparable between cases at the time of BKVN diagnosis compared to controls at the index date, except for the median daily corticosteroid dose which was significantly higher among cases (10 mg [IQR, 5–10] versus 5 mg [IQR, 0–10], *P* = 0.005) (Table 1).

All 64 cases had detectable BKV DNAemia at diagnosis, and median BK plasma viral load was 5.4 log_10_IU/ml (IQR, 4.8–6.3) versus 3.7 log_10_IU/ml (IQR, 2.7–4.8) at the time of first BKV DNAemia. The diagnosis of BKVN was biopsy-confirmed in all cases with marked viral cytopathic effect, interstitial inflammation and tubulitis, and positive SV40-LTag staining by IHC. Pathological re-examination of 52 out of the 64 diagnostic kidney biopsies for histological staging according to the ATS criteria [4] showed BKVN stage A in 13 (25%) patients, B in 37 (71%) and C in 2 (4%). BKVN was associated with significantly impaired graft function (median serum creatinine level of 205 µM [IQR, 142–269] in cases versus 126 µM [IQR, 100–159] in matched controls; *P* < 0.0001) (Table 1). The other laboratory data recorded did not differ between the two groups.

Following the diagnosis of BKVN, 95% of the cases had a reduction of the intensity of the immunosuppressive regimen except for 3 patients due to high immunological risk. More specifically, one or several immunosuppressants were changed in 53 (83%) of the patients (switch from mycophenolic acid to azathioprine in 37 [58%], and/or from tacrolimus to ciclosporin in 31 [48%]). Nineteen (30%) patients were further switched to dual therapy due to the persistence of BKV DNAemia. Twenty-one (33%) cases of BKVN received a specific therapy for the treatment of BKV infection consisting in the administration of intravenous polyvalent immunoglobulins in most cases (N = 14), cidofovir (N = 4) or leflunomide (N = 3). Overall, 31 (49%) cases had BKV DNAemia clearance in a median delay of 18 months (IQR, 7–38).

### Factors associated with biopsy-proven BKVN

In multivariate analysis, lymphopenia below 500/mm^3^ and corticosteroid daily dose above 7.5 mg were the two factors significantly associated with BKVN diagnosis (OR 3.7 [95%CI: 1.2–11.3], *P* = 0.02 and OR 3.9 [1.1–13.2], *P* = 0.03 respectively) (Table 2). No baseline characteristic or event following KT was associated with BKVN diagnosis. In particular, a c-PRA score above 15% was associated with BKVN in univariate analysis, but it was not confirmed in the multivariate model. Finally, the administration of IV polyvalent immunoglobulins was not associated with a reduced risk for BKVN.

**Table 2:** Risk factors for BKVN diagnosis

Variable	Univariate analysis		Multivariate analysis	
	Odds ratio (95% CI)	*P* (Wald’s test)	Odds ratio (95% CI)	*P* (Wald’s test)
Female sex	2.1 (0.9–5.3)	0.10	1.3 (0.2–7.1)	0.75
Prior transplant	2.3 (0.7–7.1)	0.18	1.0 (0.2–5.7)	0.98
Delayed graft function	**0.3 (0.1–0.8)**	**0.02**	0.2 (0.04–1.1)	0.06
Lymphocyte count < 500/mm^3^	**2.3 (1.1–5.1)**	**0.03**	**3.7 (1.2–11.3)**	**0.02**
c-PRA score				
15–50%	Ref.	**0.01**		0.13
< 15%	0.7 (0.3–2.0)	0.53	1.7 (0.3–9.9)	0.54
> 50%	3.0 (0.9–10.1)	0.07	6.6 (0.7–62.9)	0.10
Corticosteroid dose > 7.5 mg/day	**4 (1.5–10.7)**	**0.006**	**3.9 (1.1–13.2)**	**0.03**

*p* < 0.05 values are statistically significant and shown in bold

*BKVN* BK-virus associated nephropathy, *CI* confidence interval, *c-PRA* calculated panel reactive antibody

### Outcome and follow-up

Median duration of follow-up after BKVN diagnosis was 40 (IQR, 21–81) months for cases and 39 (IQR, 20–85) months for matched controls (*P* = 0.77, non-significant) (Table 3).

**Table 3:** Outcome and follow-up of the 64 cases of BKVN and matched controls

Variable	BKVN cases (N = 64) *n* (%); median [IQR]	Controls (N = 64) *n* (%); median [IQR]	*p*-value
Median duration of follow-up (months)	40 [21–81]	39 [20–85]	0.77
Acute rejection episodes	12 (19)	2 (3)	**0.003**
Antibody-mediated	8	2	
Cellular-mediated	2	0	
Mixed rejection	2	0	
de novo DSA (MFI > 3000)	27 (42)	16 (25)	**0.04**
Return to dialysis	22 (34)	3 (5)	** < 0.0001**
Death	4 (6)	4 (6)	1
Kidney function at last follow-up *(N* = 40*)			
Serum creatinine level (µmol/L)	214 [147–272]	139 [112–166]	**0.025**
eGFR (ml/min/1.73 m^2^)	26 [19–43]	44.5 [35–56]	0.82
Proteinuria (mg/mmol)	37 [14–74]	16 [8–30]	**0.047**

*p* < 0.05 values are statistically significant and shown in bold

*BKVN* BK-virus associated nephropathy, *DSA* donor-specific antibodies, *eGFR* estimated glomerular filtration rate (using MDRD [modification of diet in renal disease]), *IQR* interquartile range, *MFI* mean fluorescence intensity

^*^Kidney function was assessed at last follow-up only for the 40 cases (and matched controls) who were alive and had not returned to dialysis

**Fig. 1:**
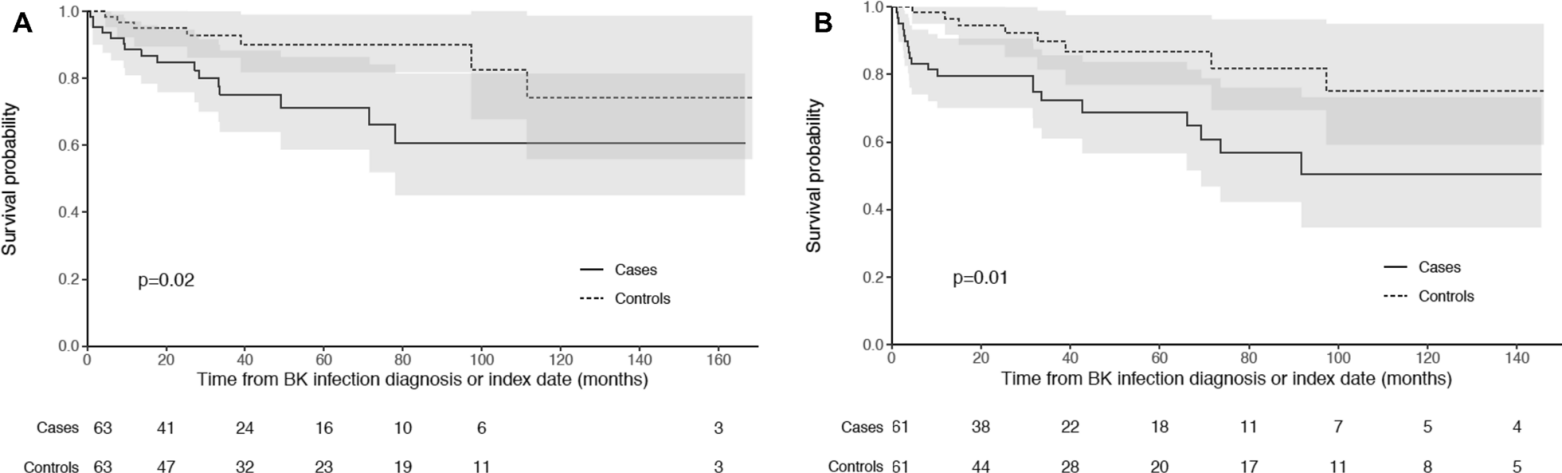
Comparative event-free survival curves between BKVN cases and matched controls for acute rejection episodes (**A**) and return to dialysis (**B**). Survival and event-free survival curves were obtained using Kaplan–Meier plots with censoring for loss to follow-up or end of observation for survival without rejection (**A**) and survival without dialysis (**B**). Comparison between cases and controls were made using log-rank tests

Following diagnosis, 12 (13.8%) cases had biopsy-proven acute rejection in a median delay of 16 (IQR, 5–37) months, compared to 2 (3%) matched controls after the index date (*P* = 0.003). The proportion of patients with significant detectable DSA (MFI > 3000) at last follow-up was also significantly higher among cases (N = 27, 42% versus N = 16, 25%; *P* = 0.04) (Table 3). Overall, survival without rejection was significantly lower among cases (*p* = 0.02) (Fig. 1A). During follow-up, 22 (34%) patients with a diagnosis of BKVN returned to dialysis in a median delay of 10 (IQR: 4–27) months, mainly due to either BKV-induced graft dysfunction (N = 16/22, 73%) or chronic humoral rejection following immunosuppression lowering (N = 4/22, 19%). Survival without return to dialysis was significantly lower among cases (*P* = 0.01) (Fig. 1B). Four (6%) patients died among both cases and controls; overall survival did not differ between the two groups (*P* = 0.98, non-significant).

At the time of last follow-up, among the 40 cases who were still alive and had not returned to dialysis, kidney function was significantly impaired compared to matched controls with higher serum creatinine (median of 214 µM [IQR, 147–272] versus 139 µM [IQR, 112–166], *P* = 0.025) and urinary protein levels (median of 37 mg/mmol [IQR, 14–74] versus 16 mg/mmol [IQR, 8–30], *P* = 0.047) (Table 3).

## Discussion

In this retrospective case–control study, we describe the clinical features and outcome of a large cohort of 64 cases of BKVN in KTR diagnosed over a 15 year-period in a French transplantation center. In our population, the frequency of BKVN was 3.7% which is similar to the one observed in other recent studies [21, 22], although it may have been underestimated. Indeed, we only included biopsy-proven cases of BKVN, and did not consider “probable” disease cases proposed recently by the BK disease Definitions Working Group of the Transplantation Associated Virus Infection Forum, and which do not require histological diagnosis [23]. Moreover, false negative BKVN biopsy rate ranges from 10 to 30% since viral-induced lesions are patchy, and some cases may be missed even though several cores are taken during the procedure.

Demographic features of our BKVN cohort did not differ from those described in previous studies regarding sex, age, or primary kidney disease distribution [24, 25]. Interestingly, the c-PRA score at baseline was significantly higher in cases compared to controls. Moreover, following KT, patients who were diagnosed BKVN had a significantly higher steroid maintenance dose, which reflects a higher level of immunosuppression. Taken together, our data suggest that patients with a high pre-transplant immunological risk should be carefully monitored for BKV infection, due to subsequent stronger immunosuppressive regimen that can favor post-transplant BKV replication and persistence due to impaired humoral and cellular immunity [26].

The median time between KT and first BKV DNAemia or BKVN diagnosis was respectively 3 and 11 months, longer than the one observed on other studies [27]. In our cohort, BKVN represented a late-onset complication occurring more than two years after KT in 11 (17%) cases. These results suggest that a prolonged screening of BKV DNAemia, longer than the first 2 years following transplantation as currently recommended in international guidelines, may be beneficial in a subset of patients. Due to delayed diagnosis, all cases presented severe graft dysfunction at the time of BKVN, with increased serum creatinine levels and extensive histological lesions on pathological examination (75% of the patients with stage B or C). Following diagnosis, we observed a lower rate of BKV DNAemia clearance compared to other studies [28]. In our population, KTR had a high immunological risk and lowering of their immunosuppressive regimen may have been delayed and/or reduced to avoid acute rejection. However, our results are concordant with previous observations showing that patients with biopsy-proven BKVN usually do not to achieve clearance rates of BKV DNAemia as high as those with probable or presumptive BKVN, as they may require more interventional steps and a longer time to recovery [9]. In accordance with international guidelines, immunosuppression tapering was first based on the switch from tacrolimus to ciclosporin A and/or MMF to azathioprine [4]. Only a small number of patients were switched to mTOR inhibitors, although the clinical efficacy of this strategy on BKV DNAemia resolution remains debated [29]. Altogether, our data reinforce the difficulties to standardize therapeutic protocols for the control of BKV infection due to the diversity of the underlying causes of nephropathy and immunization status.

Several studies have tried to identify factors associated with an increased risk for BKV infection in KTR, but only a small number have focused on biopsy-proven BKVN [18]. Moreover, interpretation and comparison of the results from these studies are difficult due to the considerable variability from one transplant center to another regarding BKV monitoring protocols, indication for kidney biopsies, and strategies for immunosuppression reduction. In this single center matched case–control study, among a large cohort of KTR with well-defined criteria for BKVN, we identify corticosteroid dose above 7.5 mg/day and lymphopenia below 500/mm^3^ as independent factors associated with BKVN diagnosis. Steroid-based immunosuppressive regimens have already been showed to be associated with the occurrence of BKV DNAemia [30, 31], and in a case–control study, prednisone dose was also shown to be a significant predictor for post-transplant BKVN [24]. Additionally, in a prospective cohort study, Hirsch and al. demonstrated that a higher steroid exposure during the first three months was independently associated with BKV replication until month 6 [13]. Tacrolimus blood levels have also been associated with an increased risk for BKV infection in a dose-dependent manner [24, 32]. In our study, we were unable to confirm this association for biopsy-proven BKVN cases due to too many missing data. However, in the absence of any standardized protocol for immunosuppression lowering in case of BKV infection, our results suggest that prednisone dose should be immediately reduced following the onset of BKV DNAemia, prior to or in combination with other modifications. Lymphopenia below 500/mm^3^ was the second factor associated with BKVN occurrence in our study. Although the multivariate model included several clinical indicators having on impact on lymphocyte count (plasma CMV reactivation or acute rejection), we cannot exclude the possibility of residual confounding factors explaining this association (such as the use of ATG or the intensification of immunotherapy). Hence, lymphopenia is a well-known factor associated with both viral and bacterial infections, and high blood lymphocyte count (CD4^+^ T > 500/mm^3^) at the time of KT and during follow-up has been showed to be protective against opportunistic infections [33]. In our study, lymphocyte count was recorded 30 days before BKVN diagnosis, making it difficult to distinguish whether the lymphopenia we observed was the cause or rather the consequence of viral infection. However, for BKV infection as for any other latent virus, T-cell response plays a key role in the control and the resolution of BKV DNAemia [34, 35], and profound lymphocyte depletion may be a be a key factor in the severity of the disease. Although we were unable to analyze CD4 + /CD8 + cell count due to too many missing data, our findings are in line with those of Schachtner et al. who showed that a loss of detectable BKV-specific T-cells as well as absolute lymphopenia (measured by CD3 + , CD4 + and CD8 + T cell counts) were highly predictive risk factors for BKV infection [36].

In our study, with a median follow up of 40 and 39 months respectively in cases and controls, we show that BKVN diagnosis was associated with a significant impact on long-term graft function, with a higher rate of return to dialysis. However, we did not observe any impact on patient survival, contrary to what was observed in another study after BKV DNAemia [37].

BKVN diagnosis was also associated with an increased incidence of acute rejection (19% versus 3%, *P* = 0.003). Allograft rejection could result both from the appearance of de novo DSA which remains a risk factor for subsequent AMR [11], but also from the inflammatory infiltrate in response to BKV cytopathic effect, and which can overlap with T-cell mediated antibody rejection [38]. Patients with late rejection diagnosis had an increased risk for graft loss, which is concordant with data from other studies [39]. Other risk factors for graft loss following BKVN have been identified such as the extant of viral burden and viremia, and the recently proposed PVN score [39–41].

Our study has a number of limitations. First, due to the relatively low number of kidney transplants performed in our center each year, cases and controls could not be paired on the basis of the number of immunosuppressive drugs, types and dosages (in addition to the other matching criteria), although this would have been most relevant. Second, we conducted a single-center study and due to homogeneous immunosuppression protocols among the study population, we could not evaluate the potential impact of the different immunosuppressants on the occurrence of BKVN. Moreover, in our cohort, most cases had an elevated c-PRA score leading physicians to slowly reduce immunosuppressants to avoid acute rejection episodes. As a consequence, MMF was not immediately put on hold and frequently replaced first by azathioprine, a practice that may differ from other centers. Overall, this limits the generalizability of our results to other transplant units using different immunosuppressive regimens, such as lower ATG doses or different antiproliferative drug use. Third, our statistical analysis lack sufficient power due to missing data in patients’ files and the small number of cases, as we included only biopsy-proven cases of BKVN. Fourth, due to the long duration of the study period, there were modifications regarding the protocols of immunosuppression and BKV monitoring. Finally, by selecting controls that never developed BKV infection during the entire observation period (rather than by the index date), the worse impact of BKVN on outcomes such as return to dialysis or graft failure this may be overestimated, due to a more ‘favorable’ control group. Complementary studies with time varying analysis on the whole cohort of KT patients could help to further explore the relationship between BKVN and clinical outcomes.

## Conclusion

In conclusion, we identify lymphopenia below 500/mm^3^ and corticosteroid maintenance therapy above 7.5 mg/day as significant risk factors for BKVN diagnosis following KT, and show a higher incidence of acute rejection and return to dialysis following BKVN. These results emphasize the need for reinforced preventive strategies for BKV detection in at-risk patients, and careful monitoring to preserve long-term graft function.

## Data Availability

The data that support the findings of this study are available on request from the corresponding author. The data are not publicly available due to privacy or ethical restrictions.

## Abbreviations

ATG: Anti-thymocyte globulins
BKV: BK virus
BKVN: BK virus associated nephropathy
CI: Confidence interval
CMV: Cytomegalovirus
CNI: Calcineurin inhibitors
c-PRA: Calculated panel reactive antigen
DNA: Deoxyribonucleic acid
DSA: Donor-specific antibodies
eGFR: Estimated glomerular filtration rate
HIV: Human immunodeficiency virus
HLA: Human leucocyte antigen
IHC: Immunohistochemistry
IQR: Interquartile range
IV: Intra-venous
KB: Kidney biopsy
KT: Kidney transplantation
KTR: Kidney transplant recipients
LTag: Large T antigen
MFI: Mean fluorescence intensity
NAbs: Neutralizing antibodies
OR: Odd ratios
qPCR: Quantitative polymerase chain reaction
SV40: Simian polyomavirus 40
VL: Viral load
